# Thermodynamic stability of the tetragonal tungsten bronze “Sr_2_NaNb_5_O_15_”

**DOI:** 10.1039/d6tc01466f

**Published:** 2026-07-22

**Authors:** Sadi Ege Parim, Thomas Brown, Teresa Roncal-Herrero, Thomas E. Hooper, Andy P. Brown, Derek C. Sinclair

**Affiliations:** a School of Chemical, Materials and Biological Engineering, University of Sheffield Sir Robert Hadfield Building, Mappin Street Sheffield S1 3JD UK separim1@sheffield.ac.uk d.c.sinclair@sheffield.ac.uk; b School of Chemical and Process Engineering, University of Leeds Leeds LS2 9JT UK

## Abstract

There has been uncertainty for several decades over the crystal chemistry, composition and thermodynamic stability of the nominally stoichiometric tetragonal tungsten bronze (TTB), Sr_2_NaNb_5_O_15_ (SNN). Recent interest in SNN-based ceramics sintered at ≥1300 °C has come about because of their high permittivity over a wide temperature range making them attractive for ‘next generation’, high temperature class II multi-layer ceramic capacitors. Here we use a solid-state reaction method between NaNbO_3_ and SrNb_2_O_6_ powders in a 1 : 2 mol ratio to verify this SNN-based TTB is not thermodynamically stable below ∼1200 °C. Instead, a mixture of A-site deficient Sr-doped NaNbO_3_ perovskite (*i.e.* Na_1−2*x*_Sr_*x*_NbO_3_ with *x* ∼ 0.20) and SrNb_2_O_6_ is obtained. We confirm the thermal instability of an A-site deficient SNN by demonstrating its complete decomposition to the same biphasic mixture at 900 °C by *in situ* powder X-ray diffraction. The local bonding arrangements and co-ordination number (CN) of the A-site cations created by the NbO_6_ octahedral arrangements play a crucial role in the thermodynamics of this system. For Sr-doped NaNbO_3_, there is a random arrangement of Na^+^, Sr^2+^ ions and vacancies on the A sites with CN < 12 in a perovskite lattice of corner sharing NbO_6_ octahedra. For SrNb_2_O_6_, the Sr^2+^ ions are in a corrugated arrangement along channels with CN = 8 based on a lattice of corner sharing Nb_2_O_10_ dimers. This phase assemblage is energetically more favourable below ∼1200 °C than Sr^2+^ and Na^+^ ions on A1 (CN = 12) and larger A2 (CN = 15) sites within a TTB lattice based on corner sharing NbO_6_ octahedra. The thermodynamic instability of the TTB-SNN below ∼1200 °C has similarities to that for the metastable TTB-PbNb_2_O_6_ and has implications for the dielectric applications of SNN ceramics.

## Abstract 2

The thermal instability of SNN across different temperatures has been investigated, revealing that both the end-member structures (NN and SN2) and the presence of A-site vacancies play significant roles.

## Graphical abstract

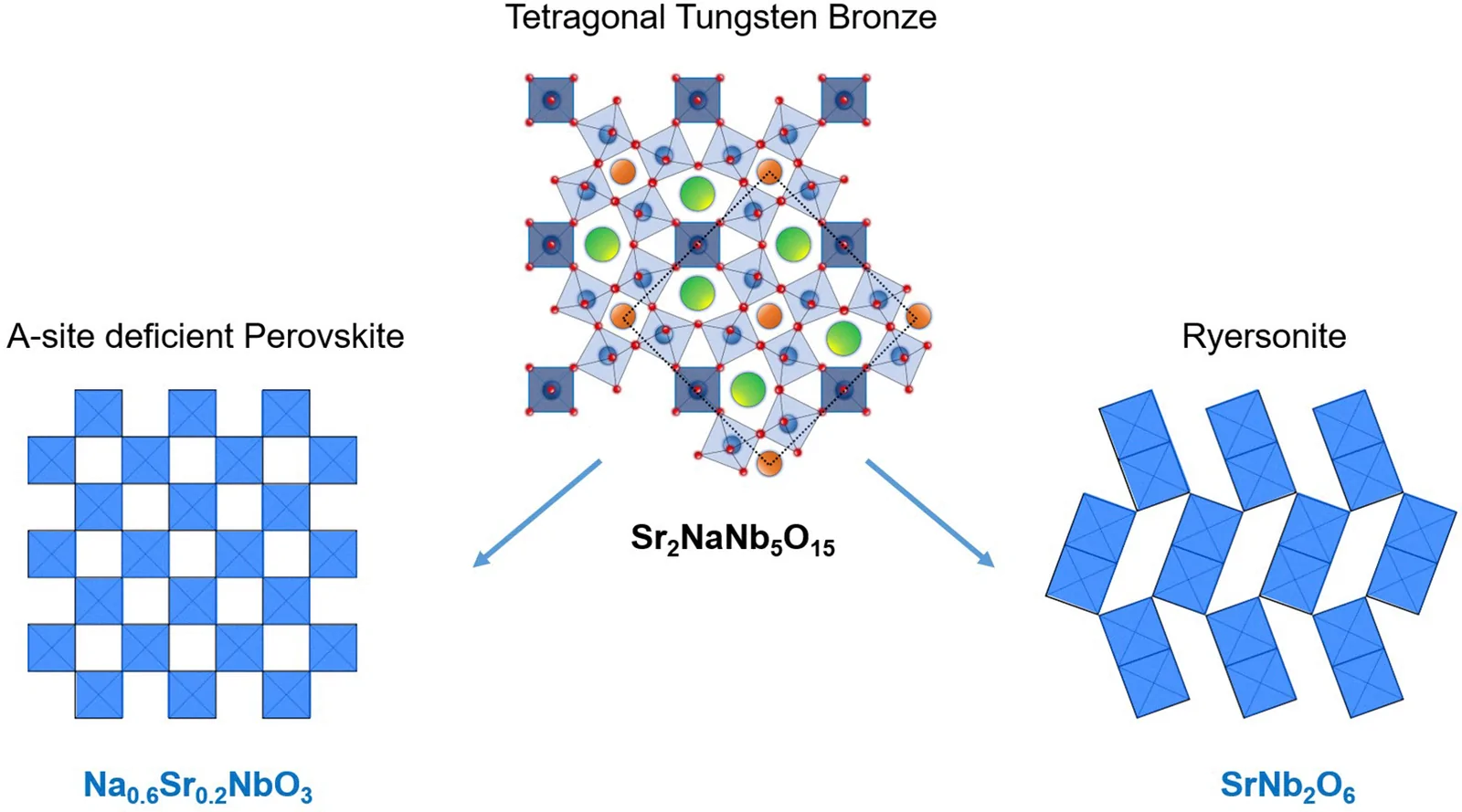

## Introduction

Tetragonal tungsten bronzes (TTBs) are an established family of polar oxides, related to ABO_3_ perovskites and have useful ferroelectric, piezoelectric and electro-optic properties. They exhibit significant structural flexibility, *e.g.* rotation and deformation of BO_6_ octahedra, A- and B-site cation displacements and complexity, *i.e.* modulated or incommensurate superstructures.^1–6^ The basic structure can be described as layers of distorted B(1)O_6_ and B(2)O_6_ corner sharing octahedra that form pentagonal (A2), square (A1) and trigonal (C) interstices which may be occupied based on the general formula (A2)_4_(A1)_2_(C)_4_(B1)_2_(B2)_8_O_30_. When all interstices are occupied, they are referred to as ‘stuffed’ TTBs; when the A sites are filled but the C sites are empty, they are ‘filled’ TTBs such as Ba_4_Na_2_Nb_10_O_30_ (more commonly written as Ba_2_NaNb_5_O_15_, BNN^7^) and when there are A-site vacancies they are ‘unfilled’ TTBs, for example Sr_*x*_Ba_1−*x*_Nb_2_O_6_.^4^ Similar to perovskites, octahedral tilting in TTBs is driven by the chemical bonding requirements of the A-cations present and is accompanied by A-site off centring whereby the size and charge of the metal ions impact the physical properties.

Despite being known for several decades,^1–6^ they continue to attract attention as dielectric materials; scientifically in terms of rationalising structure–property relations^6,8^ and technologically in the development of temperature stable^9^ and/or high energy density Class-II capacitors.^10–12^ For example, Krayzman *et al.* (2022)^8^ have studied the nanoscale structure of a classic relaxor ferroelectric (RFE) TTB (Sr_0.61_Ba_0.39_Nb_2_O_6_) exhibiting incommensurate modulation identified by a combination of local structural probes, including variable temperature neutron total scattering. They showed the modulation arises from intergrowth of structural slabs containing distinct types of BO_6_ rotations to minimise BO_6_ deformations. They also identified coupling between the polar off-centring of the symmetrically distinct Nb(1)O_6_ and Nb(2)O_6_ octahedra as a fundamental characteristic controlling the ferroelectric (FE) to RFE crossover in TTBs. This provides new understanding to the empirical crystal-chemical framework for predicting the type of octahedral rotation modulation and the accompanying conventional FE or RFE response in TTBs by Zhu *et al.* (2015).^6^

Structure–property relationships of the filled TTB phase Sr_2_NaNb_5_O_15_ have been investigated since the 1960's;^3,9,13–17^ however, the identity of the room temperature structure has been a source of controversy for many years that seems, in part, to be dependent on the synthesis and processing methods. Various orthorhombic cells have been reported for the room temperature (RT) structure based on space groups *Cmm*2,^13^*Im*2*a*^14–16^ and *P*4*bm*;^3,9,17^ however, a recent combined electron microscopy, electron, X-ray and neutron diffraction study and first principles electronic structure calculations study has shown the RT structure is best described as an incommensurate, octahedra tilted phase which approximates closely to an orthorhombic *Ama*2 phase.^18^ Based on dielectric spectroscopy (DS) measurements, Sr_2_NaNb_5_O_15_ is FE at room temperature and exhibits a sub-ambient temperature transition to a RFE (*T*_1_ ∼ −20 to −33 °C) and becomes paraelectric (PE) well above room temperature (*T*_2_ ∼ 240–305 °C). Again, it is noteworthy the values reported for *T*_1_ and *T*_2_ do vary and appear to be linked to the synthesis and processing conditions employed in sample preparation.^3,9,15,19^ There has been recent, renewed interest in the dielectric properties of Sr_2_NaNb_5_O_15_ based on lightly-doped variants (≤5 mol%) which exhibit high and stable permittivity over a very wide temperature range from −65 to 325 °C.^9^ This makes them attractive for Class II MLCCs that can operate well above market-leading BaTiO_3_ MLCCs at ∼150 °C (X8R).

**Fig. 1:**
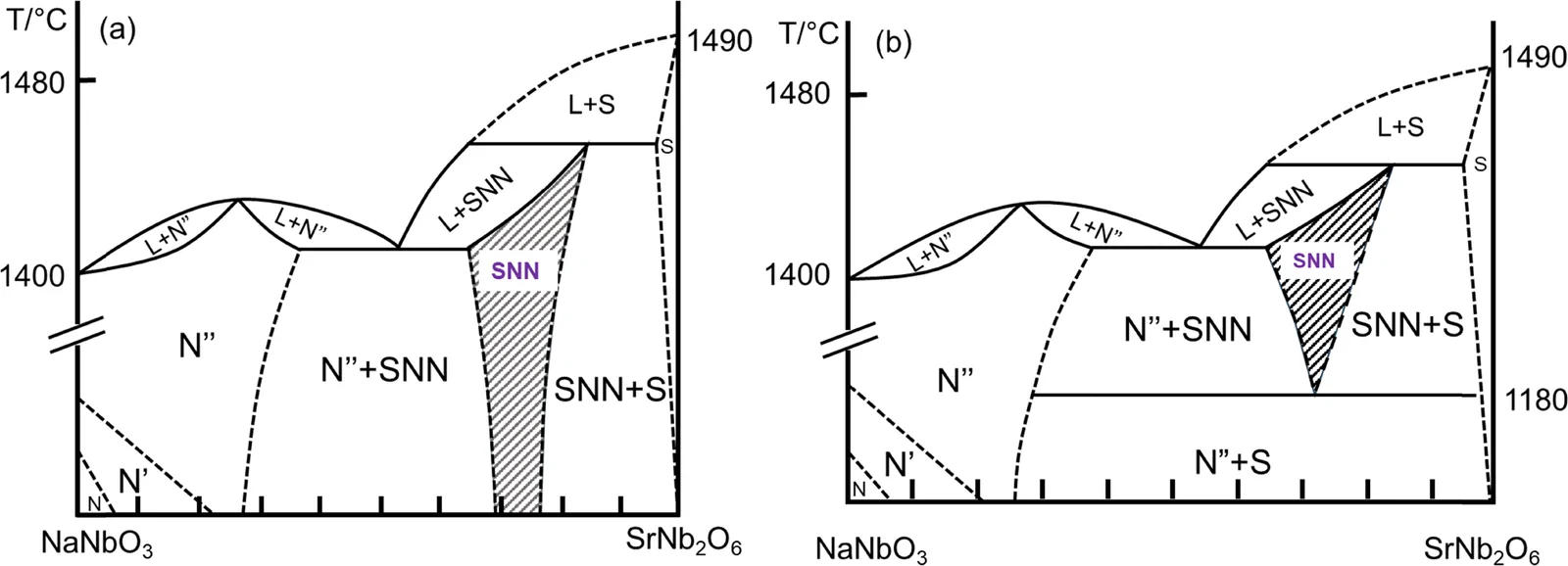
Two suggested equilibrium phase diagrams for the system NaNbO_3_ and SrNb_2_O_6_ based on different heat treatments, adapted from ref. 20. (a) A thermodynamically stable solid solution area occurs for the TTB phase, SNN and (b) shows a more limited solid solution area with a lower limit of stability for the TTB phase. N, N′ and N″ correspond to various polymorphs of Sr-doped NaNbO_3_ (perovskite), S corresponds to SrNb_2_O_6_ and L to liquid.

Several studies over the last thirty years^9,14,18^ have shown that the nominal starting composition of Sr_2_NaNb_5_O_15_ does not give rise to a single phase TTB as there is often co-formation of a low volume fraction of an additional perovskite phase based on NaNbO_3_ that is possibly Sr-doped.^15^ This implies there are A-site vacancies in the TTB phase and it is therefore non-stoichiometric or unfilled. Based on an earlier (1979) phase equilibrium study of the NaNbO_3_–SrNb_2_O_6_ system, (NN–SN2) Tang *et al.*^20^ proposed single phase, stoichiometric Sr_2_NaNb_5_O_15_ TTB at 33.3 : 66.7 NN : SN2 mol% can be obtained but only at 1415 °C. A TTB ‘unfilled’ solid solution with A-site vacancies does occur; however, the solid solution limits and stability were reported to depend on the processing conditions, Fig. 1. Samples cooled from a sintering temperature of 1300 °C for 50 hours are single phase TTB at RT with limits between ∼31 : 69 and ∼23 : 77 which correspond to a solid solution (ss) of Sr_2+*x*_Na_1−2*x*_Nb_5_O_15_ with *x* ∼ 0.07 and 0.30, respectively (SNN ss), see shaded area in Fig. 1a; however, for samples annealed between 900 and 1300 °C for 300 hours there was no evidence of any TTB phase below ∼1180 °C with the RT phase assemblage being based on a mixture of Sr-doped NN (N″, perovskite) with an A-site deficient doping mechanism of general formula Na_1−2*x*_Sr_*x*_NbO_3_ with 0 ≤ *x* ≤ 0.20^21–23^ and SN2, see Fig. 1b. The study by Tang *et al.*^20^ therefore suggests the ‘unfilled’ TTB solid solution phase with A-site vacancies (SNN ss) has a lower limit of stability below ∼1180 °C and is not thermodynamically stable at RT but to the best of our knowledge this has not been verified.

Thermodynamic instability has been reported in other TTBs, as exemplified by PbNb_2_O_6_ (PN) which displays three polymorphic forms, two of which have a TTB structure, tetragonal (t-PN) and orthorhombic (o-PN) and a third (low temperature) polymorph that is not a TTB and has a complex, layered structure, rhombohedral (r-PN). Metastable, TTB t-PN can be obtained by quenching PN from ∼1200–1250 °C.^24,25^ The t-PN polymorph is PE and (on cooling) transitions to a FE orthorhombic TTB phase with a Curie temperature of ∼570 °C.^26^ Below ∼1200–1250 °C the t-PN polymorph is, however, metastable with respect to the rhombohedral polymorph that is the thermodynamically stable, low-temperature PN phase.^24,25^ The t- to r-PN polymorphic transition is reconstructive with r-PN having a layered-type (non-TTB) structure (space group *R*3, no:146) based on edge sharing Nb_2_O_10_ octahedral dimers that have common corners to form layers with triangular and hexagonal rings of octahedra and Pb ions in the channels formed by the rings.^27^

The purpose of this study was to investigate and clarify the thermodynamic stability of the TTB phase in the NN–SN2 system based on a nominal (‘filled’) composition of Sr_2_NaNb_5_O_15_. We reacted the end members NN and SN2 in a mole ratio of 1 : 2 at various temperatures from 900 to 1200 °C to observe the phase assemblage based on laboratory XRD. A TTB phase is only observed under two heating regimes. Firstly, a dominant TTB phase is formed in samples heated at 1200 °C with the co-existence of a low volume fraction of Sr-doped NN (N″, perovskite). Secondly, the TTB phase coexists with SN2 and N″ perovskite in samples annealed at 1100 °C after being heated at 1200 °C; however, the volume fraction of the TTB decreases with increasing annealing time at 1100 °C. In addition, we show that an unfilled SNN undergoes complete decomposition at 900 °C to SN2 and N″ perovskite. The procedures are fully reversible and the TTB can be reformed by reheating powders at 1200 °C so the results are not affected by any significant Na-volatility by high temperature processing. Together these results are consistent with the phase equilibrium study by Tang *et al.*^20^ and confirm that the TTB phase that exists in the NN–SN2 system is not thermodynamically stable and has a lower limit of stability between 1100 and 1200 °C. We discuss the possible reasons for the instability of the TTB phase and the implications for its applications in Class II MLCCs.

## Experimental methods

NaNbO_3_ powder was obtained from a commercial supplier and SrNb_2_O_6_ powder was prepared by solid state reaction using starting reagents of SrCO_3_ (99.9%, Sigma Aldrich) and Nb_2_O_5_ (99.5%, H.C. Starck). The reagents were dried at 180 and 900 °C, respectively prior to batching. Powders were mixed with a 1 : 1 molar ratio and ball-milled for approximately 24 h with yttria-stabilised zirconia milling media using isopropanol as a solvent. The mixture was dried and sieved and then placed in an alumina crucible and calcined at 1100 °C for 8 h using a 5 °C min^−1^ heating rate in air. The calcined powders were then ball-milled and dried for a second time with the same process.

A ∼20 g batch of a 1 : 2 mole ratio of NaNbO_3_ and SrNb_2_O_6_ powders were ball milled for 24 h with yttria-stabilised zirconia milling media using isopropanol as a solvent. The mixture was dried and sieved and then placed in an alumina crucible and annealed in sequence at 900, 1100, 1200, 1100, 1000 and 1200 °C for 8 h using a 5 °C min^−1^ heating rate in air. Samples were annealed twice at each temperature with an intermediate ball-milling, sieving and drying step of the powders. For comparison purposes with Sr_2_NaNb_5_O_15_ ceramics reported in the literature, powder formed from the 1200 °C heat treated sample was pressed into a pellet and sintered at 1350 °C for 6 h at a heating and cooling rate of 5 °C min^−1^. The ceramic was crushed and the powder used as a representative sample of the TTB phase commonly reported for Sr_2_NaNb_5_O_15_.

Room temperature X-ray diffraction (XRD) was performed after each annealing step of the reacted powders and on the crushed ceramic using a STOE/Stadi P® diffractometer and Mo Kα radiation with *λ* = 0.7107 Å. Analysis was operated with 45 kV, 40 mA with a step size of 0.26° and count time of 90 s per step over a 2*θ* range from 6 to 42 degrees. The ICDD data base was used for phase identification.

In addition, to test the stability of Sr_2_NaNb_5_O_15_, *in situ* XRD experiments were carried out on calcined powders of Sr_2_NaNb_5_O_15_ (prepared at 1200 °C) each at 850, 900, 1000, 1100 and 1200 °C for 50 h. *In situ* X-ray diffraction was performed on a Malvern Panalytical®, Empyrean (Cu, Kα ∼1.5406 Å, 45 kV 40 mA) with an Anton Parr HTK1200 stage. An initial 30-minute room temperature scan was performed before the annealing run. Heating then proceeded to temperature at 60 °C min^−1^ and after the target temperature was reached, 10-minute scans were performed from 10 to 60° 2*θ* with step size 0.026 degrees every 10 minutes up to one hour. Following this a 30-minute scan was performed every hour up to 50 h, and then again on cooling to room temperature, with the same 2*θ* range and step size. A number of minor peaks at 25.43° 2*θ*, 35.04° 2*θ*, 37.66° 2*θ*, and 43.2° 2*θ* in some of the patterns were determined to be from the alumina sample holder.

To highlight the different radiation sources used in the STOE® (Mo-Kα) and Panalytical® (Cu-Kα) instruments for RT and *in situ* XRD data, respectively we include the instrument names and source in the relevant figure captions.

## Results and discussion

**Fig. 2:**
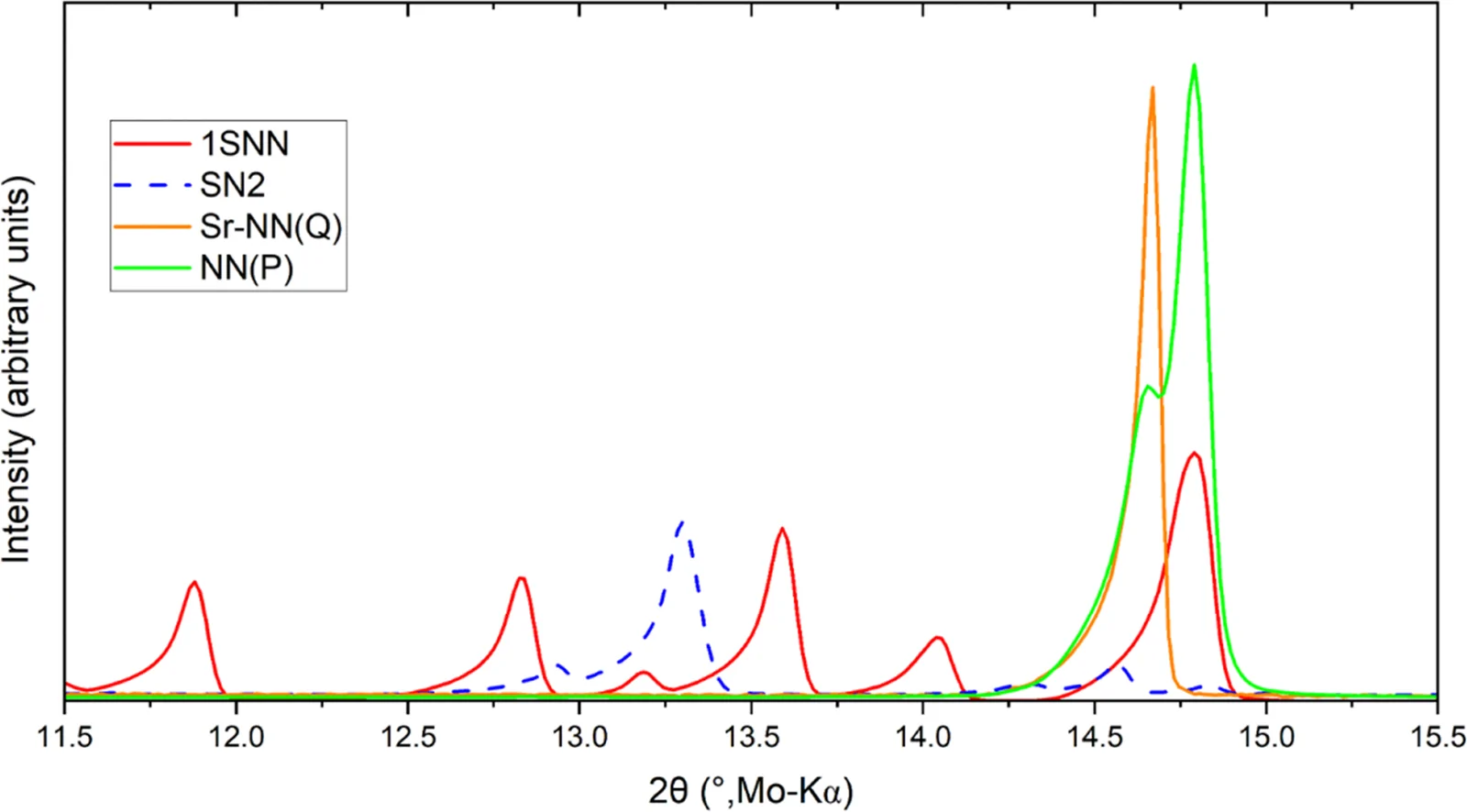
Expanded section of the STOE®RT XRD data for the TTB of nominal composition Sr_2_NaNb_5_O_15_ (1SNN, red line), SrNb_2_O_6_ (SN2, dashed blue line), Na_0.6_Sr_0.2_NbO_3_ (Sr-NN(Q), orange line) and undoped NaNbO_3_ (NN(P), green line).

RT XRD data for SN2 powder fully indexed on a monoclinic cell (*P*2_1_/*c*, *a* = 7.7217(2), *b* = 5.5937(2) and *c* = 10.9829(3) Å, beta = 90.37°).^28^ XRD data for NN powder best fitted on an orthorhombic cell based on the anti-ferroelectric P-phase (*Pbcm*, *a* = 5.5165(9), *b* = 5.5728(9) and *c* = 15.5270(3) Å). The ceramic sintered from the 1 : 2 mole ratio of NN–SN2 powders at 1350 °C was a phase mixture that was best fitted to a tetragonal TTB phase (*Pbcm a* = *b* = 12.36, *c* = 3.88 Å) with Sr-NN (N″, perovskite) as a secondary phase. Due to the close relationship between the unit cells for perovskite and TTB, it can be challenging to identify the presence and polymorph of the NN-based perovskite secondary phase in such samples. As reported in the literature,^9^ the perovskite phase is best identified as a shoulder to the left of the most intense reflection (4 2 0)/(3 1 1) for the TTB. For ease of phase identification purposes in the heat treated powders we use the strongest reflection for each phase, *i.e.* (0 3 1)/(2 1 1̄) at 13.3° for SN2, (2 0 0)/(1 1 4) at 14.8° for P-phase NN, (2 0 0)/(1 2 1) at 14.7° for Sr-NN (N″) which corresponds to *x* ∼ 0.20 in Na_1−2*x*_Sr_*x*_NbO_3_ which is a Q-phase and (4 2 0)/(3 1 1) at 14.75° for TTB, Fig. 2. Given the closeness of the strongest peaks for perovskite and TTB phases we select three additional reflections at 11.8° (3 2 0), 12.8° (2 1 1) and 13.55° (4 1 0) for the TTB phase where there are no interfering reflections for either the perovskite phase or SN2, Fig. 2.

**Fig. 3:**
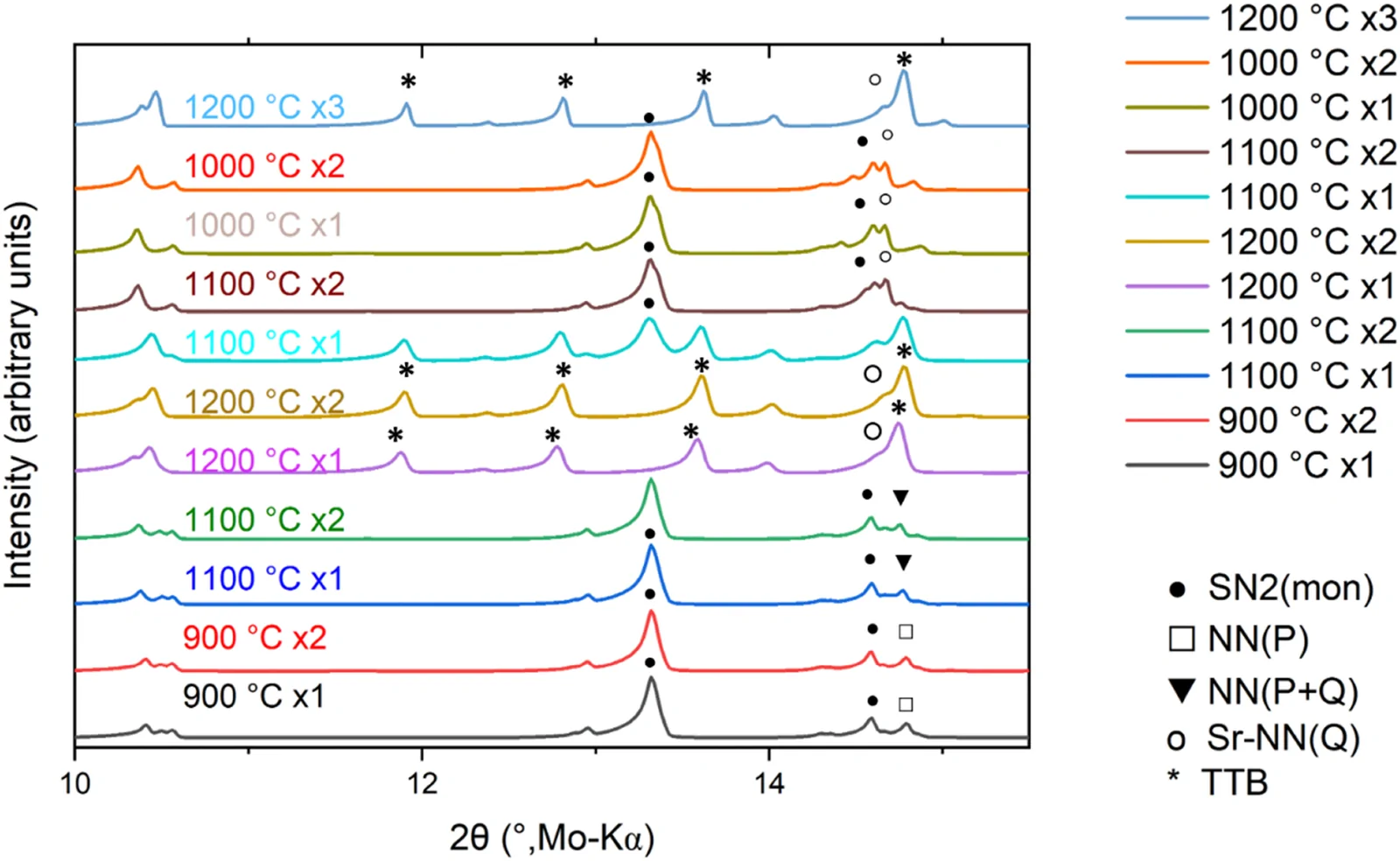
STOE® RT XRD data for a 1 : 2 mol ratio of NaNbO_3_:SrNb_2_O_6_ powders heated in air under various conditions starting from 900 °C (bottom, black line) up to 1200 °C (middle, purple line) and then down to 1000 °C (top, orange line). ×1, ×2 and ×3 indicate first, second and third anneals at the set temperature.

RT XRD traces for the heated mixtures of NN and SN2 (monoclinic, mon) after various reaction temperatures are shown in Fig. 3. There were no significant changes in the XRD patterns for both heat treatments at 900 °C and the phase assemblage remained as P-phase NN and SN2. Comparing these data with the mixed powders at RT prior to any heat treatment showed minimal changes in patterns suggesting there is negligible reaction between the end member phases at 900 °C. After heat treatment at 1100 °C there is no evidence of any TTB formation; however, reaction between the phases had started as can be evidenced by change in the XRD patterns near 14.8° where the sharp peak used to identify P-phase NN for 900 °C (open square symbol, Fig. 3) is less intense and there is significant broadening in this region with a peak developing nearer 14.7° (filled triangle symbol, Fig. 3). We interpret this as co-existence of P and Q NN phases with partial formation of the Sr-doped Na_1−2*x*_Sr_*x*_NbO_3_ perovskite solid solution with A-site vacancies. Full equilibrium has not been achieved in the mixtures but this result is consistent with the shift in XRD peaks observed for this solid solution series where samples have been equilibrated prior to XRD characterisation and indicate the P to Q phase transition to occur at *x* ∼ 0.10.^23^

**Fig. 4:**
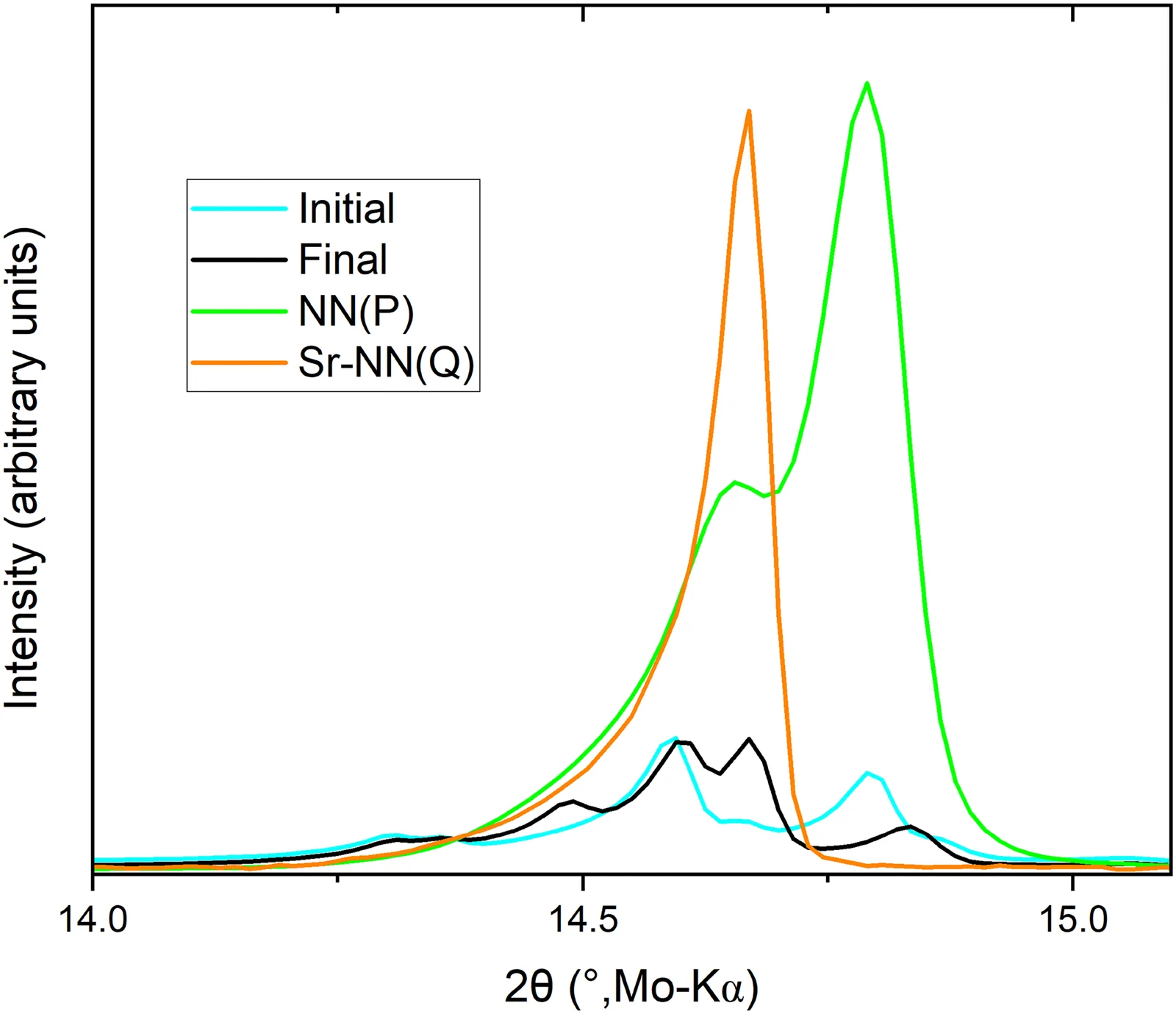
Expanded section of STOE® RT XRD data for the initial heating (900 °C ×2, blue line) and final annealing (1000 °C ×2, black line) of the 1 : 2 mol ratio of NaNbO_3_ : SrNb_2_O_6_ powders.

The TTB phase is present after heat treatment at 1200 °C (asterix symbols in Fig. 3); there is no evidence of SN2 and the NN-based perovskite is consistent with the end member Q-phase of the Sr-NN ss (open circle symbol, Fig. 3). There is minimal change in the XRD patterns between the two heat treatments at 1200 °C indicating no significant change in equilibrium between the two heat treatments at this temperature. The first heat treatment (annealing) of this 1200 °C assemblage at 1100 °C showed a clear three phase assemblage with SN2, Q-phase Sr-NNss perovskite (N″) and TTB all being present, Fig. 3. A second heat treatment at 1100 °C showed a dramatic decrease in the TTB content. These data show the TTB phase is not thermodynamically stable at 1100 °C and is decomposing into SN2 and Sr-doped NN (N″) perovskite. A final heat treatment of this mixture at 1000 °C was effective in removing the remaining TTB phase and only a biphasic mixture of SN2 and Q-phase Sr-NNss (N″) is obtained. Finally, Fig. 4 compares the initial mixture after heat treatment at 900 °C (twice) and the final mixture after 1000 °C (twice) in the range 14–15°. The peak at 14.63° associated with SN2 and has not shifted significantly, thus indicting minimal (if any) doping of Na into SN2 and is consistent with Fig. 1. In contrast, there is significant changes in the peaks associated with the perovskite phase which support that Sr has doped into the starting undoped P-phase NaNbO_3_ during the heat treatment schedule and at the end is consistent with the Q-phase (N″) of the Sr-NN ss with *x* ∼ 0.20, Fig. 4. For completeness, the final mixture after 1000 °C (twice) was reheated to 1200 °C and single phase TTB was obtained, Fig. 3 (top trace, 1200 °C x3). This showed the process was fully reversible across the annealing conditions and that any possible Na-loss due to processing at high temperatures was not responsible for the phase assemblage behaviour.

**Fig. 5:**
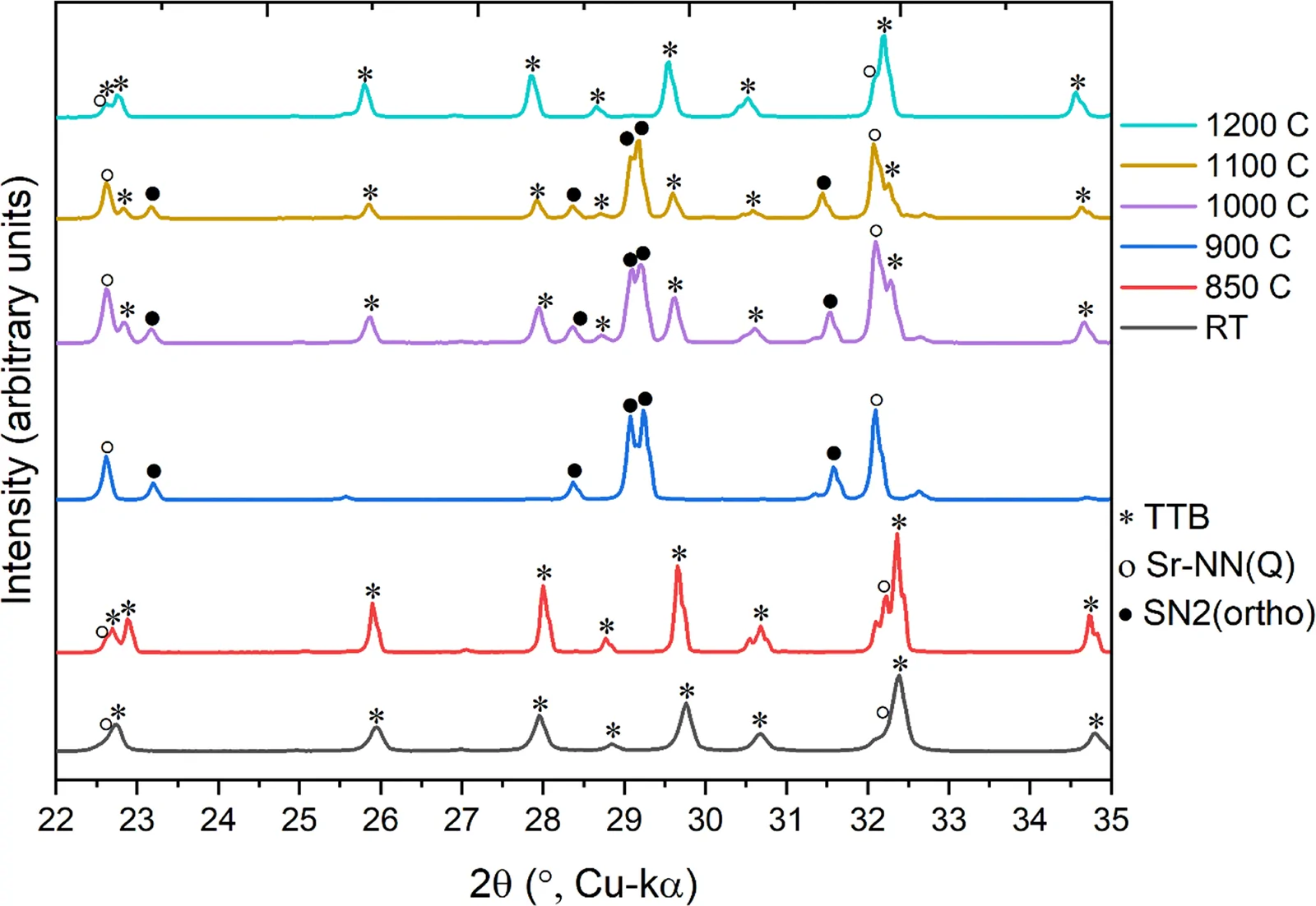
*In situ* Panalytical® XRD data (Cu-Kα) for calcined powders of Sr_2_NaNb_5_O_15_ at various temperatures. Each powder was annealed at the indicated temperature for 50 hours. The bottom trace is the RT pattern for the calcined powder.

*In situ* high temperature (HT) XRD above 800 °C was used to test the metastability of the TTB phase formed from calcined powder of Sr_2_NaNb_5_O_15_ prepared at 1200 °C and heated to various temperatures, each for 50 h, Fig. 5. The RT XRD data are included and show the calcined powder to be predominantly TTB with ∼5 wt% Q-phase Sr-NN ss. For the HTXRD data it should be noted that the TTB and SN2 phases are present as high temperature polymorphs. The TTB phase is now PE and based on a centrosymmetric tetragonal (*P*4/*mbm*) cell and SN2 is based on an orthorhombic cell (*Pnma*). These changes result in obvious splitting of the singlet peaks at ∼22.7 and ∼29° in RT XRD data into doublets in the HT XRD data for the TTB and SN, respectively. The HTXRD data show the TTB phase is retained at 850 °C but for a sample heated to 900 °C there is complete decomposition of the TTB phase and only Q-phase Sr-NN ss and SN2 (high temperature orthorhombic phase, *ortho*) are obtained. For samples heated to 1000 and 1100 °C, the HTXRD data show a three-phase mixture of TTB, Q-phase Sr-NN ss and SN2 (*ortho*) whereas for a sample heated at 1200 °C there is a return to a predominant TTB phase with Q-phase Sr-NN ss only as a minor, secondary phase again.

The *in situ* HTXRD data therefore show structural stability of the TTB phase in calcined powders of Sr_2_NaNb_5_O_15_ up to ∼850 °C when annealed for 50 h but complete decomposition at ∼900 °C. At higher temperatures (1000 and 1100 °C) there was a three-phase mixture, presumably due to the kinetics and our experiments being only for 50 h, before a return to the predominant TTB phase at 1200 °C. These results are in good agreement with phase diagram presented in Fig. 1(b).

**Fig. 6:**
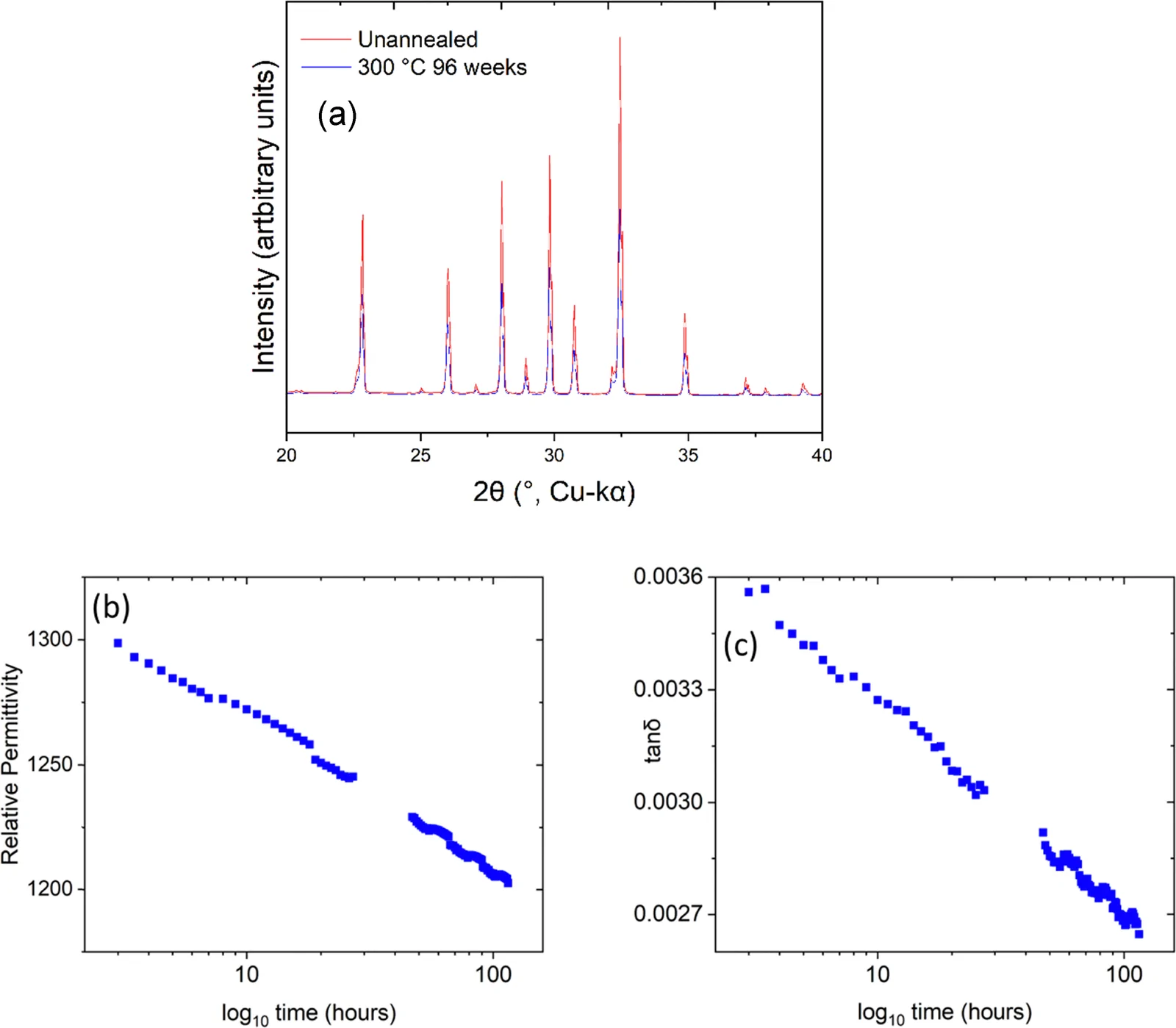
(a) Panalytical® XRD data for Sr_2_NaNb_5_O_15_ powders as prepared (red) and after being annealed at 300 °C in air for 96 weeks (blue). (b) and (c) permittivity and tan *δ* data (both 100 kHz) *versus* time at 250 °C for Sr_2_NaNb_5_O_15_ ceramics after being sintered at 1350 °C, respectively.

It is noteworthy that it is possible to obtain TTB ceramics at RT by processing powders at high temperatures (≥1300 °C) and using heating/cooling rates of 5–10 °C min^−1^, as previously reported.^9,18^ We observed no change in the RT XRD pattern for a sample that was annealed at 300 °C for 96 weeks, Fig. 6(a) which indicates that TTB decomposition at such temperatures is kinetically limited. In contrast, fixed frequency dielectric spectroscopy measurements on a ceramic held at 250 °C for ∼1000 h showed a continuous decrease in the permittivity and tan *δ* with annealing time, Fig. 6(b) and (c). It is unclear if this ageing is associated with TTB decomposition that can only be detected by the electrical measurements or if it is associated with ferroelectric domain relaxation below *T*_2_ (The Curie point). Nevertheless, these results indicate instability of the dielectric data at these temperatures clearly precludes their application as high temperature (>200 °C) dielectrics in next generation MLCCs.

**Fig. 7:**
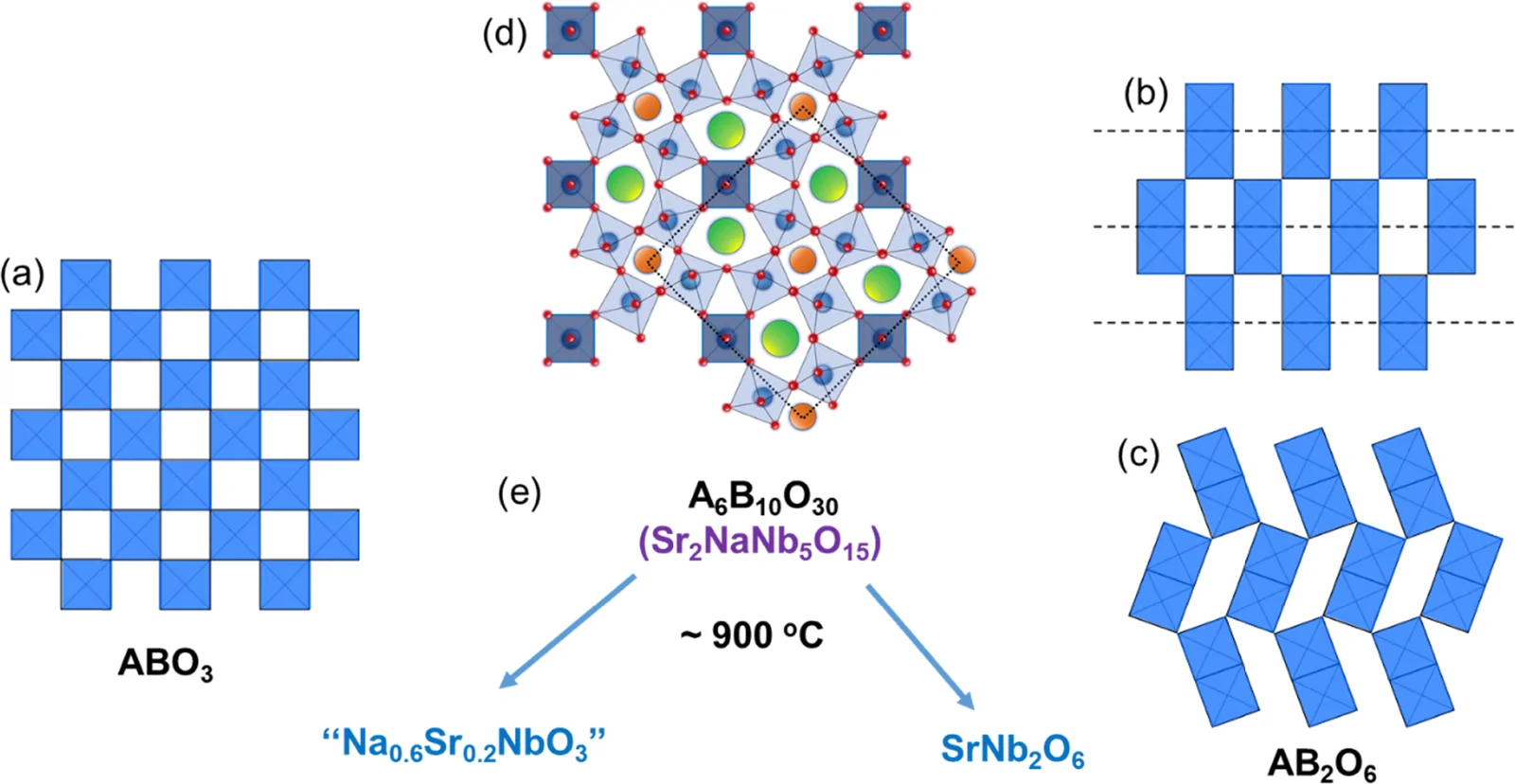
Schematic [001] projection of the coupling between corner sharing BO_6_ octahedra in (a) an ideal ABO_3_ perovskite, (b) orthorhombic AB_2_O_6_ with octahedral edge sharing dimers connected by common corners at 90° and (c) 60 and 120°. The dashed lines in (b) indicate the projections of the shear planes of perovskite blocks to create the dimers. (d) A [001] projection of the aristotype A_6_B_10_O_30_ TTB structure with tetragonal *P*4/*mbm* symmetry with the tetragonal unit cell shown by the dashed black lines. Orange, green, blue and red spheres represent A(1), A(2), B and O atoms, respectively. B(1)O_6_ and B(2)O_6_ octahedra are represented by light and dark blue shading, respectively. (e) Schematic indicting the decomposition of nominally stoichiometric Sr_2_NaNb_5_O_15_ (SNN) below ∼1200 °C into A-site deficient perovskite (∼Na_0.6_Sr_0.2_NbO_3_) and SrNb_2_O_6_.

To understand the thermodynamic instability of ‘Sr_2_NaNb_5_O_15_’ and/or the unfilled TTB ss compared to end members NN and SN2 requires consideration of the crystal structures and their interrelationships, Fig. 7. This can be done based on ideal ABO_3_ perovskite, as shown in Fig. 7a. NN is based on a perovskite structure; however, due to its tolerance factor of <1, there is significant tilting of the NbO_6_ octahedra and therefore the A-site coordination number(s) (CN) is <12. Nevertheless, it can be described as a near cubic close packed arrangement of NaO_3_ layers with Nb-ions occupying ¼ of the octahedral interstices. The NbO_6_ octahedra form a three-dimensional corner sharing network and based on a substitution mechanism of one Sr^2+^ replacing two Na^+^ ions an extensive solid solution based on A-site vacancies is created with an end member of ∼Na_0.6_Sr_0.2_NbO_3_ with ∼20% A-site vacancies, Fig. 1.^21–23^ Such a large solid solution is unsurprising given the similarity in ion size of Sr^2+^ and Na^+^ ions of 1.44 and 1.39 Å, respectively and assuming an ideal perovskite of CN = 12.

The other end member, SN2 crystallises at RT as a monoclinic, pseudo-orthorhombic modification (m-SN2) that is isostructural with orthorhombic CaTa_2_O_6_ (Rynersonite, space group *Pnma*).^29,30^ In this case, m-SN2 can be described as a framework of distorted NbO_6_ octahedra sharing edges to form Nb_2_O_10_ dimers which share common corners with Sr ions in a corrugated (zig-zag) arrangement in channels running in the direction of the *a* axis. Based on a formula of AB_2_O_6_ with a near hexagonally close packed arranged of O^2−^ ions with ½ the octahedral sites occupied by Nb, the Sr ions have a CN = 8 composed of bi-capped trigonal prismatic SrO_8_ units. The link with perovskite can be illustrated by blocks of octahedra joined as edge sharing dimers that can be created by shear planes, Fig. 7b and an orthorhombic phase produced by then rotating the blocks through angles of 60 and 120 degrees, Fig. 7c. Given the comparatively smaller ionic radius of Na^+^ compared to Sr^2+^ for CN = 8, *i.e.* 1.18 *versus* 1.40 Å, respectively, there is limited (if any) solubility of Na^+^ ions in SN2, Fig. 1.

Based on this information, it is understandable why filled Sr_2_NaNb_5_O_15_ does not exist as a single-phase material at RT and why unfilled TTB ss are metastable under certain processing conditions as shown in Fig. 1b. In an ideal filled TTB structure the smaller C-sites are empty but all the A1 (perovskite, CN = 12) and A2 (CN = 15) sites need to be occupied, Fig. 7d. A particular challenge for Sr_2_NaNb_5_O_15_ is that this combination of similar sized Sr and Na A-site cations is insufficient to satisfy the bonding requirements in the TTB structure, especially for the larger A2 sites with CN = 15 and this give rise to both non-stoichiometry and thermodynamic instability as shown in Fig. 5, where complete decomposition of the TTB phase occurs at ∼900 °C. Some replacement of the smaller Na with larger Sr ions and/or the creation of A-site vacancies in the lattice may help to partially stabilise the unfilled TTB ss structure but this requires further study. Furthermore, Tidey *et al.*^18^ used TEM to show the existence of A-site vacancies in ‘Sr_2_NaNb_5_O_15_’ but interestingly also reported some occupation of the C-sites. Presumably these sites are occupied by smaller Nb as opposed to the larger Sr and/or Na ions and further demonstrate the inability of Sr and Na to fully satisfy their bonding requirements within this TTB lattice.

There is also an interesting comparison here with PbNb_2_O_6_ and its transition from the metastable TTB t-PN to the thermodynamically stable (non-TTB) r-PN. In this case, it is not decomposition into two phases but a sluggish, reconstructive polymorphic transition into a layered crystal structure that is based on edge sharing Nb_2_O_10_ dimers that are linked by common corners and form 6- and 3-fold rings of octahedra with Pb ions in the channels formed by the rings.^27^ This AB_2_O_6_ structure is related to SrNb_2_O_6_ (Fig. 7c), in that they both form layers based around Nb_2_O_10_ dimers albeit with a more complex arrangement of the connecting octahedra in r-PN and the Pb ions in the channels. Given the A-site ions in TTB t-PN are all Pb cations, this TTB lattice has similar issues to the TTB Sr_2_NaNb_5_O_15_ where similar sized Sr and Na cations need to occupy the A1 and A2 sites. Both TTB phases exist at high temperatures where they are entropy-stabilised; however, at lower temperatures, typically <1200 °C, they are not enthalpy-stabilised and other phases are thermodynamically favoured. In the case of Sr_2_NaNb_5_O_15_ it is a phase mixture of an A-site deficient perovskite and SN2 and for PbNb_2_O_6_ it's the rhombohedral (non-TTB) polymorph as opposed to an unfilled TTB. Presumably the different arrangements of the octahedra in the two AB_2_O_6_ phases is driven by the difference in the size and bonding requirements for the Pb ions in the channels of PN as compared to Sr and Na in SNN.

Finally, a comparison of the different sequence of phase transitions in BNN^7^*versus* SNN^18^ further highlights the importance of the A-site cation sizes to not only stabilise the TTB lattice but how they influence the FE and ferroelastic properties. The large size difference of Ba and Na in BNN ensures the large A2 sites are filled by Ba with the smaller A1 sites dominated by Na. On cooling from high temperature BNN is PE (space group *P*4/*mbm*) but becomes FE (space group *P*4*bm*) at a Curie temperature of ∼580 °C with polar displacements along the *c* axis which then transitions at ∼250 °C to a lattice with both polar displacements along *c* and octahedral titling to induce ferroelastic strain (space group *Ama*2). Due to the smaller size of Sr compared with Ba and the similar size of Sr and Na ions, (on cooling) octahedral tilting occurs first (estimated >700 °C^18^) to induce ferroelastic strain in the PE state in SNN. Polar displacements to induce FE (space group *Ama*2) and RFE (space group *Aa*) phases at T_2_ and T_1_, occur at much lower temperatures of ∼300 and ∼−14 °C, respectively. The high temperature of the tilt transition in SNN therefore highlights the need for the smaller Sr and Na ions to satisfy their local bonding requirements in the TTB lattice. We also suggest that the discrepancies in the RT structure and values of *T*_1_ and *T*_2_ reported for ‘Sr_2_NaNb_5_O_15_’ in the literature are linked to sample preparation and that, in particular, the cooling rates (kinetics) from sintering temperatures of ceramics or melts for single crystals will have a significant influence on the results obtained for different compositions of the TTB ss phase and/or level of defects and the amount of decomposition that can occur.

We conclude from our annealing experiments that SNN is not thermodynamically stable below ∼1200 °C and that a system of lower energy exists based on a biphasic mixture of an A-site deficient Sr-doped NaNbO_3_ perovskite and SrNb_2_O_6_, consistent with Fig. 1b and as illustrated in Fig. 7e. In this mixture, the local bonding arrangements of the A-site cations are based on a random arrangement of Na, Sr and vacancies on A sites with CN < 12 in a perovskite lattice and with Sr ions in a corrugated arrangement along channels with CN = 8 in SrNb_2_O_6_. This is energetically more favourable than Sr and Na being in A1 (CN = 12) and A2 (CN = 15) sites within an SNN ss TTB lattice. Given the versatility of the TTB lattice it will be interesting to see if A-site doping strategies with larger and/or smaller ions will be able to thermodynamically and/or kinetically stabilise SNN-based TTB materials particularly with temperature coefficient of capacitance characteristics that can exceed X8R classification currently achievable with BaTiO_3_-based MLCCs.

## Conflicts of interest

There are no conflicts to declare.

## Data Availability

The data that support the findings of this study are available from the corresponding author, Sadi Ege Parim, upon reasonable request.

## References

[cit1] Magneli A. (1949). The crystal structure of tetragonal potassium tungsten bronze. Ark. Kemi.

[cit2] Jamieson P. B., Abrahams S. C., Bernstein J. L. (1969). ‘Ferroelectric tungsten bronze-type crystal structures. II. Barium sodium niobate Ba_(4+*x*)_Na_(2−2*x*)_Nb_10_O_30_’. J. Chem. Phys..

[cit3] Giess E. A., Scott B. A., Burns G., O’Kane D. F., Segmuller A. (1969). Alkali strontium-barium-lead niobate systems with a tungsten bronze structure: crystallographic properties and Curie points. J. Am. Ceram. Soc..

[cit4] Glass A. M. (1969). Investigation of the electrical properties of Sr_1-*x*_Ba_*x*_Nb_2_O_6_ with special reference to pyroelectric detection. J. Appl. Phys..

[cit5] Levin I., Stennett M. C., Miles G. C., Woodward D. I., West A. R., Reaney I. M. (2006). Coupling between octahedral tilting and ferroelectric order in tetragonal tungsten bronze-structured dielectrics. Appl. Phys. Lett..

[cit6] Zhu X. (2015). *et al.*, A crystal-chemical framework for relaxor versus normal ferroelectric behavior in tetragonal tungsten bronzes. Chem. Mater..

[cit7] Grendal O. G., Evans D. M., Aamlid S. S. (2023). Revisiting the structures and phase transitions of Ba_2_NaNb_5_O_15_. J. Appl. Crystallogr..

[cit8] Krayzman V., Bosak A., Playford H. Y., Ravel B., Levin I. (2022). Incommensurate modulation and competing ferroelectric/antiferroelectric modes in tetragonal tungsten bronzes. Chem. Mater..

[cit9] Brown T. (2021). *et al.*, New high temperature dielectrics: Bi-free tungsten bronze ceramics with stable permittivity over a very wide temperature range. J. Eur. Ceram. Soc..

[cit10] Gao Y. (2024). *et al.*, Optimizing high-temperature energy storage in tungsten bronze-structured ceramics via high-entropy strategy and bandgap engineering. Nat. Commun..

[cit11] Duan J. (2024). *et al.*, High-Entropy Tungsten Bronze Ceramics for Large Capacitive Energy Storage with Near-Zero Losses. Adv. Funct. Mater..

[cit12] Wang Z., Li D., Liu W., He L., Xu D., Liu J., Ren J., Wang X., Liu Y., He G., Bao J., Fang Z., Yan G., Liang X., Zhou T., Zhao W., Liu W., Wang D., Zhou D. (2025). Ultra-high energy storage in lead-free NaNbO_3_-based relaxor ceramics with directional slush-like polar structure design. Nat. Commun..

[cit13] Ravez J., Budin J.-P., Hagenmuller P. (1972). Etude comparative des proprietes cristallographiques, dielectriques et d’optique non lineaire des phases ABCNb_5_O_15_ (A = Ca, Sr, Ba, B = Ca, Sr, Ba, C = Na, K) de type “bronzes oxygenes de tungstene quadratiques. J. Solid State Chem..

[cit14] García-Gonzalez E., Torres-Pardo A., Jimenez R., Gonzalez-Calbet J. M. (2007). Structural singularities in ferroelectric Sr_2_NaNb_5_O_15_. Chem. Mater..

[cit15] Torres-Pardo A., Jimenez R., Gonzalez-Calbet J. M., Garcia-Gonzalez E. (2011). Structural effects behind the low temperature nonconventional relaxor behavior of the Sr_2_NaNb_5_O_15_ bronze. Inorg. Chem..

[cit16] Xu S. (2022). *et al.*, Enhanced energy storage properties and superior thermal stability in SNN-based tungsten bronze ceramics through substitutional strategy. J. Eur. Ceram. Soc..

[cit17] Lanfredi S., Genova D. H. M., Brito I. A. O., Lima A. R. F., Nobre M. A. L. (2011). Structural characterization and Curie temperature determination of a sodium strontium niobate ferroelectric nanostructured powder. J. Solid State Chem..

[cit18] Tidey J. P. (2024). *et al.*, Structural origins of the dielectric anomalies in the filled tetragonal tungsten bronze Sr_2_NaNb_5_O_15_. Commun. Mater..

[cit19] Zhang T.-H., Liu Y.-G., Zhao J.-Q., Huang Z.-H., Fang M.-H. (2015). Effects of atmospheric powder on dielectric, piezoelectric, and ferroelectric properties of NaSr_2_Nb_5_O_15_ ceramics. Int. J. Appl. Ceram. Technol..

[cit20] Tang D.-S., Liang J.-K., Shi T.-J., Zhang Y.-L., Tian J.-H., Li W.-H. (1979). Investigation of the pseudo ternary system SrNb_2_O_6_-NaNbO_3_-LiNbO_3_. Acta Phys. Sin..

[cit21] Torres-Pardo A., Jiminez R., Gonzalez-Calbet J. M., Garcia-Gonzalez E. (2008). Room temperature ferroelectric in Na_1-*x*_Sr_*x*/2_□_*x*/2_NbO_3_ through the introduction of cationic vacancies. Chem. Mater..

[cit22] Torres-Pardo A., Jiminez R., Gonzalez-Calbet J. M., Garcia-Gonzalez E. (2009). Induction of relaxor behaviour in Na_1-*x*_Sr_*x*/2_□_*x*/2_NbO_3_ through the introduction of cationic vacancies. Chem. Mater..

[cit23] Hooper T. E., Sinclair D. C. (2024). Structural, dielectric and conduction behaviour of A-site deficient Sr_*x*_Na_1-2*x*_NbO_3_ ceramics. J. Mater. Chem. C.

[cit24] Francombe M. H. (1956). Structural, dielectric and optical properties of ferroelectric lead metaniobate. Acta Crystallogr..

[cit25] Lee H. S., Kimura T. (1998). Effects of microstructure on the dielectric properties and piezoelectric properties of lead metaniobate. J. Am. Ceram. Soc..

[cit26] Goodman G. J. (1953). Ferroelectric properties of lead metaniobate. J. Am. Ceram. Soc..

[cit27] Olsen G. H., Sorby M. H., Hauback B. C., Selbach S. M., Grande T. (2014). Revisiting the crystal structure of rhombohedral lead metaniobate. Inorg. Chem..

[cit28] Marinder B. O., Wang P. L., Werner P. E. (1986). Powder diffraction studies of SrNb_2_O_6_ and SrNb_6_O_16_. Acta Chem. Scand., Ser. A.

[cit29] Beck H. P. (2013). A study on AB_2_O_6_ compounds, Part III: Co-ordination needs and patterns of connectivity. Z. Kristallogr..

[cit30] Jahnberg L. (1963). Crystal Structure of Orthorhombic CaTa_2_O_6_. Acta Chem. Scand..

